# Comparative screening performance and multicenter validation of novel inflammatory biomarkers (NLR, SII, and CAR) for risk stratification of diabetic foot ulcer in patients with type 2 diabetes mellitus

**DOI:** 10.3389/fmed.2026.1890603

**Published:** 2026-09-08

**Authors:** Yue-feng He, Yue Zhou, Jian-gang Lu

**Affiliations:** 1 Graduate Training Base of Jinzhou Medical University (Chongqing University Fuling Hospital), Chongqing, China; 2 Department of Endocrinology and Metabolism, Dujiangyan People’s Hospital, Chengdu, Sichuan, China; 3 Department of Critical Care Medicine, Dujiangyan People’s Hospital, Chengdu, Sichuan, China; 4 Department of Endocrinology, Chongqing Municipal Key Clinical Specialty, Chongqing University Fuling Hospital, Fuling, China

**Keywords:** C-reactive protein-to-albumin ratio, diabetic foot ulcer, neutrophil-to-lymphocyte ratio, systemic immune-inflammation index, type 2 diabetes mellitus

## Abstract

**Objective:**

To compare the screening performance of the neutrophil-to-lymphocyte ratio (NLR), systemic immune-inflammation index (SII), and C-reactive protein-to-albumin ratio (CAR) for risk stratification of diabetic foot ulcer (DFU) in patients with type 2 diabetes mellitus (T2DM), and to develop an integrated risk screening model with clinically applicable cutoff values.

**Methods:**

This multicenter retrospective cross-sectional study included hospitalized patients with T2DM from Dujiangyan People's Hospital and Chongqing University Fuling Hospital between January 2024 and December 2025. Demographic characteristics, clinical variables, and laboratory parameters were collected, and NLR, SII, and CAR were calculated. Univariate and multivariate logistic regression analyses were performed to identify risk factors associated with DFU, and these results were combined with random forest analysis for variable selection. Four risk screening models were subsequently constructed. Model discrimination was evaluated using receiver operating characteristic (ROC) curves, area under the curve (AUC), and DeLong's test. Optimal cutoff values were determined using the Youden index. Model stability was assessed using bootstrap resampling (1000 iterations). Calibration performance was evaluated using the Hosmer–Lemeshow test and calibration curves, while clinical utility was assessed by decision curve analysis (DCA). A prespecified sensitivity analysis excluding patients with moderate-to-severe DFU infection was performed. The Fuling cohort served as an independent external validation cohort, with the development-derived threshold fixed and applied without recalibration.

**Results:**

A total of 962 patients from the Dujiangyan cohort (161 with DFU, 16.7%) and 600 patients from the Fuling cohort (204 with DFU, 34.0%) were included. Multivariate logistic regression analysis demonstrated that CAR was significantly associated with DFU in both cohorts [Dujiangyan cohort: odds ratio (OR) per 0.1-unit increase = 1.029, 95% *confidence interval (CI)*: 1.017–1.041, *P* < 0.001; Fuling cohort: OR = 1.013, 95% *CI*: 1.001–1.025, *P* = 0.031]. The integrated risk model (Model 4) achieved an AUC of 0.84 (95% *CI*: 0.80–0.87) in internal validation, with a bias-corrected C-index of 0.83 (95% *CI*: 0.80–0.87) and good calibration (Hosmer–Lemeshow *P* = 0.866). In the external validation cohort, Model 4 achieved an AUC of 0.61 (95% *CI*: 0.56–0.66) using the fixed development-derived threshold (0.136). However, a prespecified sensitivity analysis excluding patients with moderate-to-severe DFU infection substantially attenuated the CAR association in both cohorts, indicating that CAR's performance is largely driven by acute infection-related inflammation.

**Conclusion:**

CAR is independently associated with prevalent DFU in hospitalized patients with T2DM, but its association is largely driven by acute infection-related inflammation. The integrated risk model demonstrated good internal discrimination and modest external performance, and may assist in cross-sectional risk stratification for hospitalized patients; however, local recalibration is recommended before clinical deployment, and prospective studies in non-infected populations are needed before broader implementation.

## Introduction

Diabetic foot ulcer (DFU) is one of the most severe chronic complications of type 2 diabetes mellitus (T2DM). Despite substantial advances in overall diabetes management in recent years, the incidence and disease burden of DFU remain persistently high. According to the International Diabetes Federation (IDF Diabetes Atlas, 2025), diabetes has become one of the leading causes of non-traumatic lower-limb amputation worldwide (1). Previous studies have reported that the lifetime risk of developing foot ulcers in patients with diabetes ranges from approximately 19% to 34% (2). A considerable proportion of these patients ultimately progress to amputation, which is associated with a markedly increased long-term mortality risk (3). Although vascular reconstruction and wound management techniques have continuously improved, early identification and risk stratification of DFU remain major clinical challenges.

With increasing patient age and prolonged disease duration, an increasingly recognized phenomenon is that glycemic control alone cannot fully prevent the development of DFU. The existence of this “residual risk” suggests that pathological mechanisms beyond metabolic dysregulation are involved in disease progression. Recent evidence has consistently demonstrated that chronic low-grade inflammation plays a pivotal role in the development and progression of T2DM and its complications (4). Some studies have further proposed that patients with T2DM may experience a persistent inflammatory state associated with inflammaging and metaflammation (5). This sustained inflammatory environment may gradually impair endothelial function, promote atherosclerotic progression, and disrupt immune responses and tissue repair processes, thereby increasing susceptibility to DFU. Therefore, biomarkers reflecting this inflammatory status may have potential value for the early identification of high-risk individuals.

Against this background, several inflammation-related indices derived from routine blood tests have attracted increasing attention. The neutrophil-to-lymphocyte ratio (NLR) was among the earliest proposed biomarkers and has been shown to partially reflect alterations in immune status. Previous studies have demonstrated its association with disease severity in cardiovascular disorders and diabetes-related infections (6, 7). The systemic immune-inflammation index (SII), which incorporates platelet parameters in addition to NLR, may more comprehensively reflect inflammatory activation and thrombotic tendency (8). In addition, the C-reactive protein-to-albumin ratio (CAR), integrating both inflammatory burden and nutritional status, has been suggested to provide a more comprehensive representation of the overall physiological condition of patients with chronic diseases and has demonstrated prognostic value across multiple disease settings (9).

However, current evidence regarding these biomarkers in diabetes-related complications remains limited. In particular, most DFU-related studies have been based on relatively small sample sizes and single-center populations, and the comparative screening value among these biomarkers has not been systematically evaluated. Moreover, studies integrating inflammatory biomarkers with clinical characteristics for comprehensive risk stratification are still lacking. Therefore, a systematic comparison of the screening performance of NLR, SII, and CAR for DFU risk based on real-world data is warranted, together with further exploration of their clinical utility in risk stratification.

Given the retrospective cross-sectional design, we recognize that the observed associations may be influenced by reverse causation—elevated inflammatory markers could result from the ulcer itself rather than precede its development. Accordingly, the present multicenter retrospective cross-sectional study was designed to systematically compare the screening performance of NLR, SII, and CAR for DFU in patients with T2DM, and to develop an integrated risk stratification model incorporating clinical variables. Internal and independent external validation were performed to evaluate the stability and generalizability of the model. This study aims to provide clinicians with a low-cost and readily accessible “inflammation–nutrition” dual-risk stratification tool for identifying hospitalized patients at higher risk of prevalent DFU, thereby facilitating timely diagnostic evaluation and comprehensive risk assessment.

## Methods

### Study design and population

This multicenter retrospective cross-sectional study included hospitalized patients with T2DM who were admitted to Dujiangyan People's Hospital and Chongqing University Fuling Hospital between January 1, 2024, and December 31, 2025. All patients were diagnosed with T2DM according to the criteria established by the World Health Organization (WHO) or the American Diabetes Association (ADA) (10). DFU was diagnosed according to the International Working Group on the Diabetic Foot (IWGDF) guidelines, which define it as skin breakdown or ulceration occurring distal to the ankle joint. For patients with DFU, the severity of foot infection was graded using the IWGDF/Infectious Diseases Society of America (IDSA) classification (Grade 1: uninfected; Grade 2: mild; Grade 3: moderate; Grade 4: severe) (11).

This study was approved by the Ethics Committees of both participating centers (Dujiangyan People's Hospital approval No. 2025-S-32; Chongqing University Fuling Hospital approval No. 2026CDFSFLYYEC-049). Because of the retrospective design and anonymized data processing, the requirement for informed consent was waived.

#### Inclusion criteria

1. Age ≥18 years;
2. Confirmed diagnosis of T2DM;
3. Complete clinical and laboratory data are available during hospitalization.

#### Exclusion criteria

1. Presence of malignant tumors or severe acute infections unrelated to DFU;
2. Missing data exceeding 30% for key variables;
3. Repeated hospital admissions (only the first hospitalization record was included).

#### Data collection and variable definitions

Baseline patient data were extracted from the hospital electronic medical record systems. All blood samples were collected within 24 h of admission and prior to any debridement or wound manipulation. All variables were obtained from the first recorded values within 24 h after admission.

### Demographic and clinical variables

Demographic and clinical information included age, sex, height, body weight, and body mass index (BMI). Previous medical histories, including hypertension, coronary heart disease, cerebrovascular disease, peripheral vascular disease (PVD), and neuropathy, were recorded. Lifestyle-related information, such as smoking history and medication use, including hypoglycemic agents, was also collected.

### Diagnostic criteria for neuropathy and PVD

#### Neuropathy

Neuropathy was diagnosed based on nerve conduction velocity (NCV) testing results. In clinical research, NCV is widely regarded as the gold standard for diagnosing diabetic peripheral neuropathy (DPN) (12). Examinations were performed by neurologists according to standardized procedures, including assessment of at least the sural nerve (sensory), tibial nerve, and peroneal nerve (motor). Diabetic peripheral neuropathy was diagnosed when abnormalities in conduction velocity (slowing) or amplitude reduction were identified in any tested nerve. These examinations were conducted systematically in all patients regardless of DFU status.

### Peripheral vascular disease

PVD was diagnosed using the ankle-brachial index (ABI) or vascular ultrasonography. According to the American College of Cardiology/American Heart Association (ACC/AHA) guideline-defined criteria for peripheral artery disease (PAD), an ABI ≤0.90 was considered diagnostic ^(^13). Alternatively, vascular ultrasonography demonstrating lower-extremity arterial atherosclerotic plaque formation or stenosis (luminal stenosis ≥50%) was also considered diagnostic (14). Patients meeting either criterion were classified as having PVD. These assessments were performed systematically in all patients, irrespective of DFU status.

### Vital signs

Vital signs included systolic blood pressure, diastolic blood pressure, heart rate, body temperature, and respiratory rate.

### Laboratory parameters

Laboratory measurements included routine blood tests (white blood cell count, neutrophil count, lymphocyte count, hemoglobin level, and platelet count), biochemical parameters (creatinine, blood urea nitrogen, albumin, cholesterol, and triglycerides), and C-reactive protein (CRP).

### Calculation of inflammatory indices

The inflammatory indices were calculated as follows:

Neutrophil-to-lymphocyte ratio (NLR) = neutrophil count/lymphocyte count (15);
Systemic immune-inflammation index (SII) = platelet count  ×  neutrophil count/lymphocyte count (16);
C-reactive protein-to-albumin ratio (CAR) = CRP/albumin (9).

### Outcome definition

The primary outcome was the presence or absence of DFU.

#### Statistical analysis

All statistical analyses were performed using R software (version 4.3.1). Continuous variables were expressed as median (interquartile range, IQR), and comparisons between groups were conducted using the Mann–Whitney U test. Categorical variables were presented as frequency (percentage) and compared using the chi-square test or Fisher's exact test, as appropriate. All statistical tests were two-sided, and a *P* value <0.05 was considered statistically significant.

All variables had complete data for the primary outcome and key model predictors (age, sex, smoking history, creatinine, lymphocyte count, and CAR components after calculation). Missingness was observed in several auxiliary variables [height, weight, glycated hemoglobin (HbA1c), CRP, cholesterol, and albumin]; the variable “coronary heart disease” was excluded because its missing rate exceeded 30% in the Fuling cohort. For the remaining missing data, multiple imputation by chained equations was performed to generate twenty complete datasets (m = 20), with the outcome variable included in the imputation model, separately for each cohort. Regression coefficients and performance measures were pooled across imputed datasets using Rubin's rules.

Potential DFU-related variables were screened using univariate logistic regression analysis (*P* < 0.05), followed by random forest (ntree=1,000, mtry≈18, nodesize=5) and least absolute shrinkage and selection operator (LASSO) regression with 10-fold cross-validation (*λ*.1se criterion) for variable selection. Multivariate logistic regression models were then constructed, with multicollinearity assessed using variance inflation factors (VIF) (VIF < 5). The events-per-variable ratio was calculated to assess model parsimony. The linearity assumption for continuous variables was verified using restricted cubic splines. The scaling rationale for continuous variables (e.g., CAR per 0.1, creatinine per 10) is detailed in Supplementary Table S3.

Model discrimination was evaluated using receiver operating characteristic (ROC) curves, area under the curve (AUC), and DeLong's test. Optimal cutoff values were determined using the Youden index. Calibration was assessed using the Hosmer–Lemeshow test, calibration slope, calibration-in-the-large, and calibration curves, with Brier scores calculated for overall accuracy. Bootstrap resampling (1,000 iterations) was performed for internal validation, with variable selection and coefficient estimation repeated in each iteration. Clinical utility was evaluated using decision curve analysis (DCA).

A prespecified sensitivity analysis excluding patients with moderate-to-severe DFU infection (IWGDF/IDSA Grade 3 or 4) was performed to assess the impact of infection-related inflammation.

Data from Dujiangyan People's Hospital were used for model development and internal validation, whereas data from Chongqing University Fuling Hospital served as the independent external validation cohort. For external validation, the development-derived optimal cutoff was fixed and applied directly without recalibration.

## Results

### Baseline characteristics

The baseline analysis included 962 patients with T2DM from Dujiangyan People's Hospital, among whom 161 patients developed DFU. Compared with the non-DFU group, patients in the DFU group were older (71.0 vs. 63.0 years, *P* < 0.01), had lower body weight (61.0 vs. 63.0 kg, *P* = 0.04), higher heart rate (84 vs. 80 bpm, *P* < 0.01), and lower diastolic blood pressure (71 vs. 75 mmHg, *P* < 0.01).

Regarding inflammation-related biomarkers, NLR, SII, and CAR were all significantly elevated in the DFU group (all *P* < 0.01). In addition, patients with DFU showed higher proportions of smoking history, coronary heart disease, and coronary artery stenosis (all *P* < 0.01). Laboratory findings further demonstrated increased white blood cell count, neutrophil percentage, blood urea nitrogen, creatinine, and CRP levels, whereas hemoglobin and albumin levels were significantly lower in the DFU group (all *P* < 0.01).

Overall, patients with DFU exhibited more pronounced inflammatory activation, poorer nutritional status, and a greater burden of comorbidities (Table 1).

**Table 1:** Baseline characteristics of study population.

Variable	ALL (*n* = 962)	No DFU (*n* = 801)	DFU (*n* = 161)	H/*χ*^2^	*P*-value
NLR	3.58 (2.38–6.66)	3.37 (2.31–5.84)	5.81 (3.53–9.56)	48.71	<0.01
SII	583.62 (371.90–1099.42)	539.59 (358.23–957.60)	1001.00 (528.66–1908.71)	36.41	<0.01
CAR	0.25 (0.23–0.31)	0.24 (0.22–0.28)	0.42 (0.25–1.32)	80.52	<0.01
Sex (Male/Female)	519 (53.95%)/443 (46.05%)	435 (54.31%)/366 (45.69%)	84 (52.17%)/77 (47.83%)	0.25	0.67
Age, years	65.00 (54.00–75.00)	63.00 (53.00–74.00)	71.00 (60.00–78.00)	29.67	<0.01
Weight, kg	63.00 (54.00–71.00)	63.00 (55.00–72.00)	61.00 (52.00–69.00)	4.11	0.04
Height, cm	160.00 (155.00–169.00)	160.00 (155.00–169.00)	160.00 (155.00–170.00)	0.6	0.44
Heart rate, bpm	80.00 (73.00–89.75)	80.00 (73.00–88.00)	84.00 (74.00–96.00)	10.3	<0.01
Body temperature, °C	36.50 (36.40–36.60)	36.50 (36.40–36.60)	36.50 (36.40–36.70)	0.43	0.51
Systolic BP, mmHg	127.00 (118.00–140.00)	126.00 (118.00–140.00)	130.00 (118.00–140.00)	1.55	0.21
Diastolic BP, mmHg	74.50 (68.00–83.00)	75.00 (68.00–85.00)	71.00 (66.00–80.00)	14.02	<0.01
Respiration rate	20.00 (20.00–20.00)	20.00 (20.00–20.00)	20.00 (20.00–20.00)	2.17	0.14
Anticoagulant use (Yes/No)	461 (47.92%)/501 (52.08%)	376 (46.94%)/425 (53.06%)	85 (52.80%)/76 (47.20%)	1.84	0.2
Hypoglycemic drugs (Yes/No)	949 (98.65%)/13 (1.35%)	798 (99.63%)/3 (0.37%)	151 (93.79%)/10 (6.21%)	34.26	<0.01
Smoking history (Yes/No)	69 (7.17%)/893 (92.83%)	18 (2.25%)/783 (97.75%)	51 (31.68%)/110 (68.32%)	174.38	<0.01
Drug allergy history (Yes/No)	4 (0.42%)/958 (99.58%)	3 (0.37%)/798 (99.63%)	1 (0.62%)/160 (99.38%)	0.2	1
Peripheral vascular disease (Yes/No)	766 (79.63%)/196 (20.37%)	605 (75.53%)/196 (24.47%)	161 (100.00%)/0 (0.00%)	49.48	<0.01
Neuropathy (Yes/No)	597 (62.06%)/365 (37.94%)	439 (54.81%)/362 (45.19%)	158 (98.14%)/3 (1.86%)	106.89	<0.01
Nephropathy (Yes/No)	248 (25.78%)/714 (74.22%)	197 (24.59%)/604 (75.41%)	51 (31.68%)/110 (68.32%)	3.51	0.08
Retinopathy (Yes/No)	69 (7.17%)/893 (92.83%)	53 (6.62%)/748 (93.38%)	16 (9.94%)/145 (90.06%)	2.22	0.19
Hypertension (Yes/No)	485 (50.42%)/477 (49.58%)	409 (51.06%)/392 (48.94%)	76 (47.20%)/85 (52.80%)	0.8	0.39
Coronary heart disease (Yes/No)	122 (12.68%)/840 (87.32%)	89 (11.11%)/712 (88.89%)	33 (20.50%)/128 (79.50%)	10.66	<0.01
Cerebrovascular disease (Yes/No)	137 (14.24%)/825 (85.76%)	121 (15.11%)/680 (84.89%)	16 (9.94%)/145 (90.06%)	2.93	0.10
Cerebrovascular stenosis (Yes/No)	119 (12.37%)/843 (87.63%)	113 (14.11%)/688 (85.89%)	6 (3.73%)/155 (96.27%)	13.33	<0.01
Coronary artery stenosis (Yes/No)	21 (2.18%)/941 (97.82%)	0 (0.00%)/801 (100.00%)	21 (13.04%)/140 (86.96%)	106.81	<0.01
WBC, ×10⁹/L	6.42 (5.13–8.61)	6.30 (5.08–8.22)	7.30 (5.92–9.96)	21.05	<0.01
Neutrophil, %	72.10 (64.50–80.85)	71.00 (63.50–79.40)	77.80 (70.60–84.70)	36.56	<0.01
Neutrophil absolute, ×10⁹/L	4.62 (3.39–6.43)	4.43 (3.31–6.09)	5.60 (4.15–8.48)	30.08	<0.01
Lymphocytes, ×10⁹/L	1.21 (0.87–1.61)	1.23 (0.90–1.63)	1.03 (0.77–1.40)	16.43	<0.01
RBC, ×10^12^/L	4.40 (3.94–4.85)	4.49 (4.04–4.91)	4.01 (3.43–4.38)	65.23	<0.01
Hemoglobin, g/L	134.00 (119.00–149.00)	137.00 (122.00–150.00)	121.00 (104.00–132.00)	77.01	<0.01
Platelets, ×10⁹/L	164.00 (128.00–204.00)	163.00 (128.00–203.00)	168.00 (124.00–213.00)	0.28	0.59
Glucose, mmol/L	12.06 (7.98–17.99)	12.32 (8.15–18.42)	11.51 (7.61–16.17)	4.49	0.03
ALT, U/L	22.00 (16.00–32.00)	22.00 (16.00–33.00)	19.00 (14.00–31.00)	7.23	0.01
AST, U/L	21.00 (17.00–29.00)	21.00 (17.00–28.00)	22.00 (17.00–33.00)	2.29	0.13
Total bilirubin, μmol/L	12.91 (9.85–17.29)	13.21 (10.08–17.60)	11.67 (8.57–15.50)	16.66	<0.01
Albumin, g/L	41.30 (38.30–44.50)	42.00 (39.10–44.80)	37.10 (32.60–41.10)	97.06	<0.01
BUN, mmol/L	6.46 (5.13–8.51)	6.30 (5.03–8.14)	8.17 (5.93–10.95)	38.32	<0.01
Creatinine, μmol/L	67.52 (55.80–88.63)	65.79 (54.80–83.66)	83.50 (62.10–120.64)	36.08	<0.01
eGFR, mL/min/1.73m^2^	97.20 (82.25–106.88)	98.60 (84.10–108.00)	89.60 (73.20–99.90)	27.81	<0.01
LDL, mmol/L	2.41 (1.86–3.00)	2.48 (1.96–3.07)	2.01 (1.47–2.55)	45.06	<0.01
HDL, mmol/L	1.24 (1.02–1.53)	1.26 (1.03–1.54)	1.18 (0.96–1.41)	6.03	0.01
HbA1c, %	8.85 (7.20–11.20)	9.00 (7.20–11.20)	8.10 (6.90–11.00)	3.17	0.08
Cholesterol, mmol/L	4.33 (3.57–5.13)	4.43 (3.71–5.23)	3.75 (3.06–4.38)	59.53	<0.01
Triglycerides, mmol/L	1.87 (1.24–3.07)	2.03 (1.31–3.24)	1.42 (1.00–2.06)	37.68	<0.01
Monocyte, %	5.50 (4.50–6.70)	5.50 (4.40–6.60)	5.70 (4.70–6.80)	2.21	0.14
CRP, mg/L	10.00 (10.00–11.19)	10.00 (10.00–10.00)	15.83 (10.00–48.31)	75.2	<0.01

Data are presented as median IQR for continuous variables and number (percentage, %) for categorical variables. Comparisons between groups were performed using the Mann–Whitney U test for continuous variables and the χ^2^ test or Fisher's exact test for categorical variables, as appropriate. DFU, diabetic foot ulcer; NLR, neutrophil-to-lymphocyte ratio; SII, systemic immune-inflammation index; CAR, C-reactive protein-to-albumin ratio; BP, blood pressure; WBC, white blood cell; RBC, red blood cell; ALT, alanine aminotransferase; AST, aspartate aminotransferase; BUN, blood urea nitrogen; eGFR, estimated glomerular filtration rate; LDL, low-density lipoprotein; HDL, high-density lipoprotein; HbA1c, glycated hemoglobin; CRP, C-reactive protein. Wagner classification, tissue loss grading, and foot infection grading were used to assess the severity of diabetic foot. A two-sided *P* value <0.05 was considered statistically significant.

#### Variable selection and construction of predictive risk models based on machine learning and regression analysis

Univariate analysis demonstrated that NLR, SII, CAR, smoking history, neuropathy, coronary heart disease, creatinine, albumin, cholesterol, and several other clinical characteristics were significantly associated with DFU occurrence (all *P* < 0.05). Subsequent random forest analysis (parameters: ntree=1,000, mtry≈18, nodesize=5) further identified CAR, NLR, SII, age, smoking history, creatinine, cholesterol, and lymphocyte count as variables with high predictive importance within the model. Collinearity diagnostics revealed no evident multicollinearity among the included variables (VIF < 5).

Notably, due to quasi-complete separation in the DFU group (all 161 patients had PVD, and 158 had neuropathy), these two variables were excluded from the final multivariable models to ensure stable coefficient estimates. Multivariate logistic regression analysis confirmed that, after adjustment for potential confounding factors, CAR remained significantly associated with DFU in the Dujiangyan cohort [odds ratio (OR) per 0.1-unit increase = 1.029, 95% CI: 1.017–1.041, *P* < 0.001], and in the Fuling cohort (OR = 1.013, 95% CI: 1.001–1.025, *P* = 0.031) (Table 2). In the Dujiangyan cohort, 63.4% of DFU patients had moderate-to-severe infection (IWGDF/IDSA Grade 2–3), compared with 35.3% in the Fuling cohort. In a prespecified sensitivity analysis excluding patients with moderate-to-severe DFU infection, the CAR association was substantially attenuated in Dujiangyan (OR dropped to 1.162, *P* = 0.12) and became null in Fuling (OR = 0.984, *P* = 0.84), indicating that CAR's strong performance is largely driven by acute infection-related inflammation (Supplementary Table S1).

**Table 2:** Multivariable logistic regression analysis of risk factors for DFU in the training and validation cohorts.

Variable	Unit	Dujiangyan (Training, *n* = 962)		Fuling (Validation, *n* = 600)	
		OR (95% CI)	*P*-value	OR (95% CI)	*P*-value
**Model 1**					
NLR	per 1	1.018 (0.999–1.036)	0.055	1.032 (1.011–1.057)	0.005
Age	per 1 yr	1.038 (1.024–1.053)	<0.001	1.015 (1.000–1.030)	0.056
Sex (male vs. female)	male vs. female	0.841 (0.588–1.200)	0.340	0.914 (0.634–1.312)	0.626
**Model 2**					
SII	per 100	1.008 (0.999–1.016)	0.072	1.015 (1.007–1.026)	0.002
Age	per 1 yr	1.039 (1.025–1.054)	<0.001	1.015 (1.000–1.030)	0.058
Sex (male vs. female)	male vs. female	0.836 (0.585–1.193)	0.325	0.894 (0.620–1.285)	0.548
**Model 3**					
CAR	per 0.1	1.035 (1.024–1.045)	<0.001	1.012 (1.000–1.024)	0.049
Age	per 1 yr	1.038 (1.023–1.054)	<0.001	1.015 (1.000–1.030)	0.056
Sex (male vs. female)	male vs. female	0.850 (0.589–1.224)	0.384	0.877 (0.609–1.257)	0.477
**Model 4**					
CAR	per 0.1	1.029 (1.017–1.041)	<0.001	1.013 (1.001–1.025)	0.031
Age	per 1 yr	1.031 (1.013–1.049)	<0.001	1.013 (0.997–1.028)	0.109
Sex (male vs. female)	male vs. female	1.406 (0.920–2.163)	0.117	0.678 (0.445–1.031)	0.070
Creatinine	per 10	1.045 (1.018–1.077)	0.002	1.005 (0.997–1.014)	0.240
Cholesterol	per 1	0.637 (0.520–0.772)	<0.001	—	—
Lymphocyte count	per 1	0.938 (0.650–1.322)	0.724	1.176 (1.074–1.339)	0.003
Smoking history	yes vs. no	24.432 (13.000–48.000)	<0.001	0.578 (0.375–0.886)	0.012

OR, odds ratio; CI, confidence interval; NLR, neutrophil-to-lymphocyte ratio; SII, systemic immune-inflammation index; CAR, C-reactive protein-to-albumin ratio. Model 4a (Dujiangyan) included CAR + age + sex + creatinine + cholesterol + lymphocyte count + smoking history (7 variables). Model 4b (Fuling) included CAR + age + sex + creatinine + lymphocyte count + smoking history (6 variables; cholesterol unavailable). Neuropathy and PVD were excluded due to quasi-complete separation (all DFU patients had PVD; 158/161 had neuropathy). ORs for continuous variables are scaled to clinically meaningful units (see Supplementary Table S3 for scaling rationale). —, not applicable.

Based on the statistical screening results and the variable importance ranking derived from the random forest analysis, the final integrated risk screening model, Model 4a (full, 7 variables: CAR + sex + age + creatinine + cholesterol + lymphocyte count + smoking history), was developed for the Dujiangyan cohort. For the Fuling external validation cohort, a reduced Model 4b (6 variables, excluding cholesterol due to data unavailability) was applied.

#### Predictive discrimination and validation of the models

**Figure 1:**
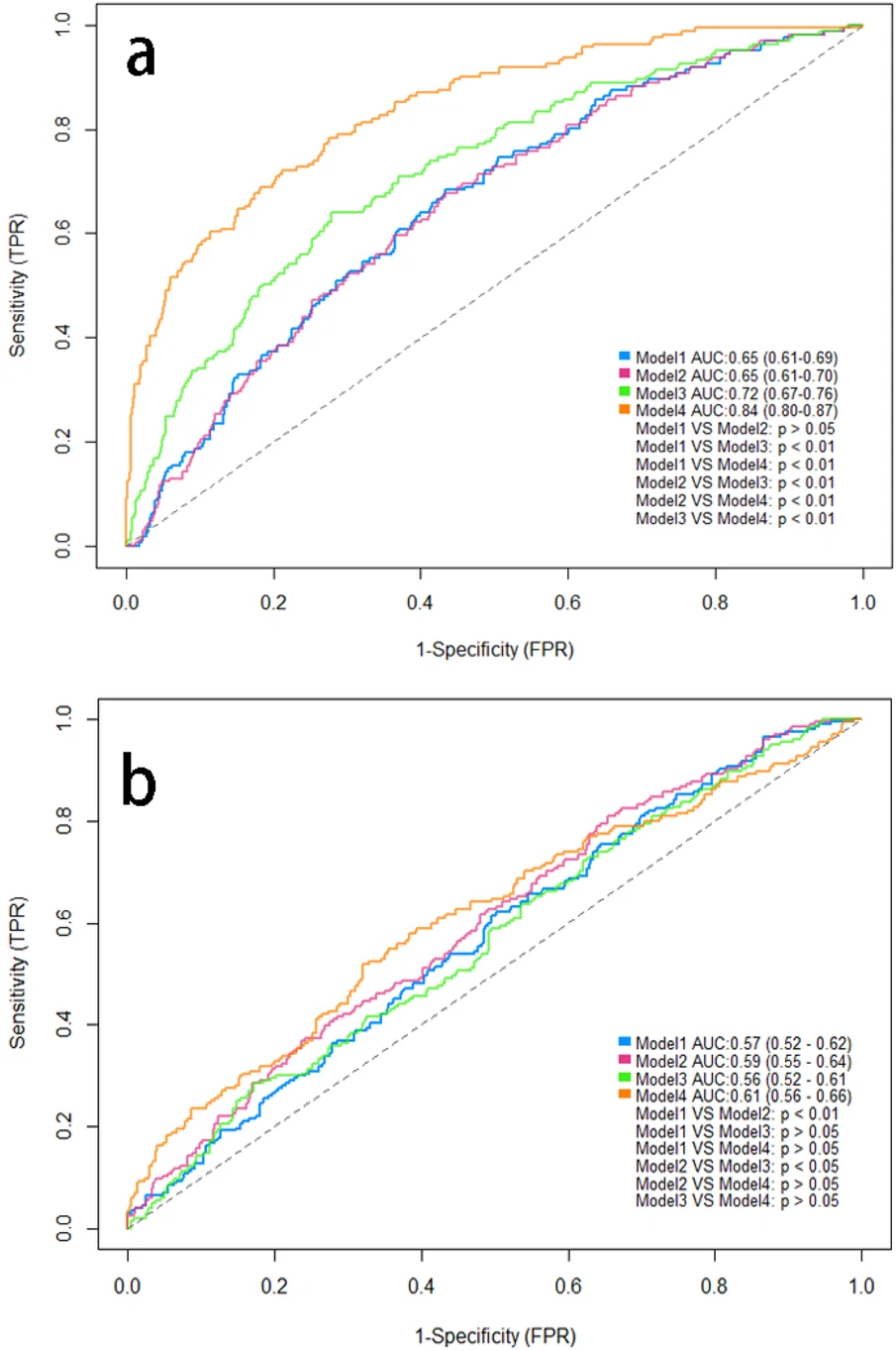
Receiver operating characteristic (ROC) curves of the four predictive models in the internal **(a)** and external **(b)** validation cohorts. **(a)** In the Dujiangyan cohort (internal validation), Model 4 demonstrated the highest AUC and significantly outperformed the other three models (all *P* < 0.01). **(b)** In the Fuling cohort (external validation), Model 4 achieved the numerically highest AUC (0.61), but DeLong tests revealed no statistically significant differences between Model 4 and Model 1 (*P* > 0.05), Model 2 (*P* > 0.05), or Model 3 (*P* > 0.05). The area under the ROC curve (AUC) reflects the ability of each model to discriminate between patients with and without DFU. AUC values with 95% confidence intervals (CIs) are presented in the figure. Pairwise comparisons of AUCs were performed using DeLong's test. Model 4 (orange line) showed the best discriminative performance in the internal cohort; in the external cohort, it maintained the numerically highest AUC, although the differences from the other models were not statistically significant.

ROC curve analysis demonstrated that Model 4 achieved the highest discriminative performance in both cohorts (Table 3 and Figure 1).

**Table 3:** Predictive performance of the four models in the training and validation cohorts.

Model	Dujiangyan (Training, *n* = 962)				Fuling (Validation, *n* = 600)			
	AUC (95% CI)	Cut-off	Sens.	FPR	AUC (95% CI)	Fixed Cut-off	Sens.	FPR
Model 1	0.650 (0.610–0.690)	0.160	0.680	0.430	0.570 (0.520–0.620)	0.160	0.620	0.500
Model 2	0.650 (0.610–0.700)	0.170	0.680	0.430	0.590 (0.550–0.640)	0.170	0.810	0.650
Model 3	0.720 (0.670–0.760)	0.180	0.640	0.280	0.560 (0.520–0.610)	0.180	0.280	0.170
Model 4	0.840 (0.800–0.870)	0.156	0.720	0.212	0.610 (0.560–0.660)	0.136	0.662	0.619

AUC, area under the curve; CI, confidence interval; Sens., sensitivity; FPR, false-positive rate (1−specificity). Cut-off values were determined using the Youden index in the training cohort. For external validation, the development-derived cut-off was fixed and applied directly to the Fuling cohort without recalibration. Model 4 corresponds to Model 4a (7 variables) in Dujiangyan and Model 4b (6 variables) in Fuling. Internal validation using bootstrap resampling (1,000 iterations, with variable selection repeated in each iteration) yielded a bias-corrected C-index of 0.83 (95% CI: 0.80–0.87) for Model 4a.

In the Dujiangyan cohort, Model 4a achieved an AUC of 0.84 (95% CI: 0.80–0.87), which was significantly superior to Model 1 (0.65), Model 2 (0.65), and Model 3 (0.72) (DeLong test, all *P* < 0.01). According to the Youden index, the optimal cutoff value for Model 4a was 0.156, corresponding to a sensitivity of 0.72 and a false-positive rate of 0.212.

Internal validation using bootstrap resampling (1,000 iterations, with variable selection repeated in each iteration) yielded a bias-corrected C-index of 0.83 (95% CI: 0.80–0.87), confirming excellent model stability without obvious overfitting (events-per-variable ratio = 23:1).

In the external validation cohort (Fuling cohort), Model 4b maintained relatively favorable predictive performance, with an AUC of 0.61 (95% CI: 0.56–0.66). Although Model 2 showed the highest AUC among the single-biomarker models (0.59), DeLong tests revealed no statistically significant difference between Model 4 and Model 1 (*P* > 0.05), Model 2 (*P* > 0.05), or Model 3 (*P* > 0.05).

To address the threshold heterogeneity concern, we fixed the internal cutoff of Model 4b (0.136) and applied it directly to the Fuling cohort without recalibration. At this fixed threshold, the sensitivity was 0.662, specificity 0.381, PPV 0.355, and NPV 0.686. The externally re-optimized cutoff (0.140) yielded nearly identical operating characteristics (sensitivity 0.647, specificity 0.404), suggesting that the observed cutoff drift is primarily due to differences in population prevalence (16.7% vs. 34.0%) rather than model failure.

We further quantified the incremental value of CAR over a Base clinical model (age + sex + smoking history + creatinine). In the internal cohort, adding CAR significantly improved the AUC from 0.816 to 0.838 (DeLong *P* = 0.004), whereas adding NLR or SII did not. However, in the external cohort, CAR showed no significant incremental value (*P* = 0.232), whereas albumin alone significantly improved the model (AUC = 0.666, *P* < 0.001) (Table 4).

**Table 4:** Incremental value of inflammatory biomarkers added to the base clinical model.

Cohort/Model	AUC (95% CI)	Brier score	Likelihood Ratio Test (*P*)	DeLong (*P*)
Internal (Dujiangyan, *n* = 962)				
Base clinical *	0.816 (0.780–0.852)	0.1,019	(ref)	—
Base clinical + NLR	0.816 (0.780–0.852)	0.1019	0.818	0.836
Base clinical + SII	0.817 (0.781–0.852)	0.1019	0.631	0.441
Base clinical + CAR	0.838 (0.803–0.872)	0.0979	<0.001	0.004
Base clinical + CRP	0.834 (0.799–0.868)	0.0995	<0.001	0.011
Base clinical + Albumin	0.849 (0.815–0.883)	0.0927	<0.001	0.013
External (Fuling, *n* = 600)				
Base clinical *	0.597 (0.549–0.646)	0.2144	(ref)	—
Base clinical + NLR	0.624 (0.576–0.672)	0.2089	<0.001	0.076
Base clinical + SII	0.648 (0.601–0.695)	0.2074	<0.001	0.004
Base clinical + CAR	0.611 (0.563–0.660)	0.2127	0.033	0.232
Base clinical + CRP	0.603 (0.554–0.652)	0.2140	0.286	0.421
Base clinical + Albumin	0.666 (0.621–0.711)	0.2056	0.003	<0.001

AUC, area under the curve; CI, confidence interval; NLR, neutrophil-to-lymphocyte ratio; SII, systemic immune-inflammation index; CAR, C-reactive protein-to-albumin ratio; CRP, C-reactive protein. Base clinical model: age + sex + smoking history + creatinine (plus cholesterol in the Dujiangyan cohort; cholesterol was not available in Fuling). The Base model corresponds to the clinical variables included in Model 4a/4b excluding the inflammatory biomarker.

#### Model calibration and clinical utility

**Figure 2:**
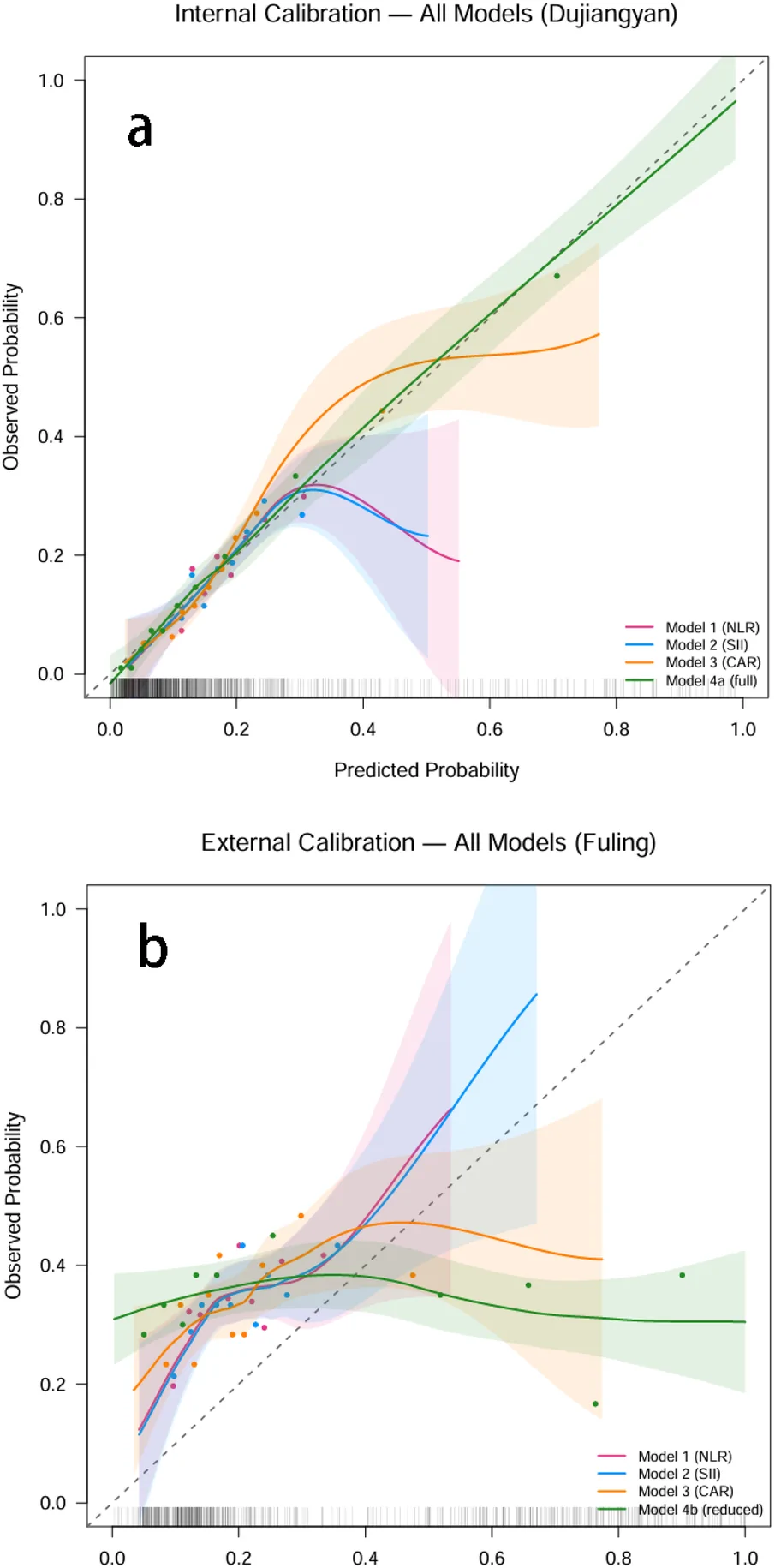
Calibration curves of the four predictive models in the internal **(a)** and external **(b)** validation cohorts. The *x*-axis represents the predicted probability of DFU, and the *y*-axis represents the observed probability. The diagonal dashed line indicates ideal calibration. **(a)** In the internal cohort, Model 4 (orange line) showed the closest agreement with the ideal line, indicating excellent calibration performance. **(b)** In the external cohort, all models showed substantial miscalibration when applied without recalibration, with Model 4 exhibiting marked deviation from ideal calibration (calibration slope = −0.0277; see Supplementary Table S2).

Calibration curve analysis (Figure 2) and the Hosmer–Lemeshow test demonstrated excellent internal calibration for Model 4a in the development cohort (*χ*^2^ = 3.9, *P* = 0.866), with a calibration slope of 1.000 and a calibration-in-the-large of −0.000, confirming near-perfect agreement between predicted and observed probabilities in the training set (Supplementary Table S2). The Brier score for Model 4a in internal validation was 0.0979.

However, marked calibration deterioration was observed in the external validation cohort. When Model 4b was directly applied to the Fuling cohort without recalibration, the calibration slope dropped to −0.0277 and the calibration-in-the-large shifted to −0.6841, with a highly significant Hosmer–Lemeshow test (*χ*^2^ = 533.9, *P* < 0.001), indicating substantial miscalibration (Supplementary Table S2). This external miscalibration, despite modest rank-order discrimination (AUC = 0.61), is primarily attributable to marked differences in baseline DFU prevalence (16.7% vs. 34.0%) and case-mix heterogeneity between the two centers, and underscores the need for population-specific intercept recalibration before clinical deployment.

**Figure 3:**
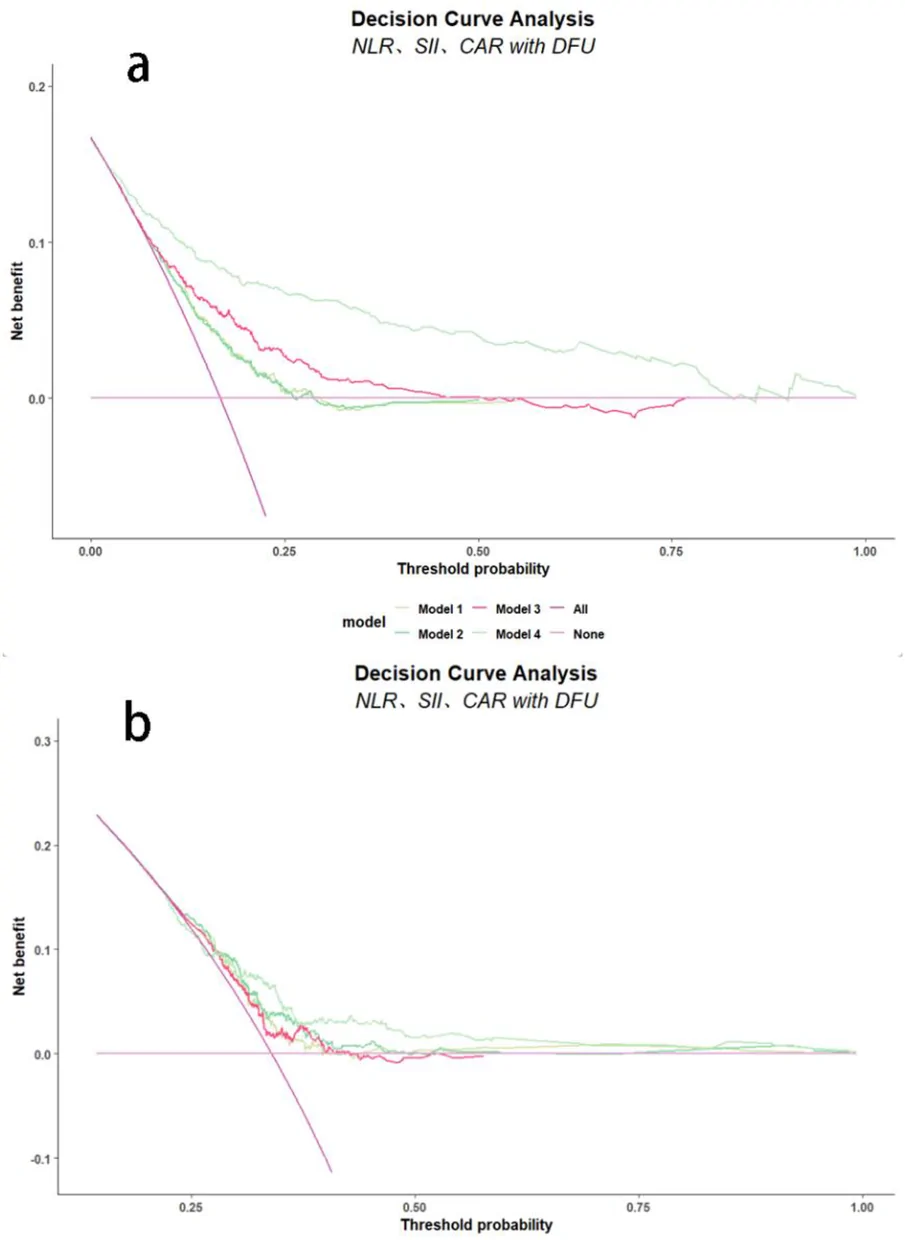
Decision curve analysis (DCA) of the four predictive models in the internal **(a)** and external **(b)** validation cohorts. Decision curve analysis illustrates the clinical utility of each model across a range of threshold probabilities. The “None” strategy (purple line) assumes that no patients develop DFU, whereas the “All” strategy (gray line) assumes that all patients develop DFU.

DCA (Figure 3) demonstrated that, in the internal validation cohort (Dujiangyan cohort), Model 4 consistently achieved greater clinical net benefit than the other models as well as the “treat-all” and “treat-none” strategies across a broad threshold probability range (approximately 0.05–0.80).

Similarly, in the external validation cohort (Fuling cohort), Model 4 demonstrated comparable or slightly improved net clinical benefit compared with the other models across the major clinical decision-making range (approximately 0.05–0.60), and generally outperformed Model 3, which showed negative net benefit at certain threshold probabilities. These findings suggest that Model 4 maintains reasonable clinical utility across different patient populations, although local recalibration is recommended before deployment.

## Discussion

The present multicenter real-world study systematically evaluated the screening value of inflammation-related biomarkers for DFU risk in patients with T2DM. The findings demonstrated that the NLR, SII, and CAR were all significantly associated with prevalent DFU. Among these biomarkers, CAR exhibited the most robust and consistent independent association after multivariable adjustment and external validation. These findings highlight the important role of systemic inflammation and nutritional imbalance in the pathogenesis of DFU and further suggest that composite biomarkers integrating multidimensional pathological information may outperform single inflammatory indicators.

The development of DFU results from the combined effects of long-term metabolic dysregulation and impaired host defense mechanisms. Diabetes-related metabolic abnormalities contribute to sensory loss and foot deformities, rendering the foot vulnerable to repetitive mechanical injury. Under such conditions, inflammatory and tissue repair responses are often insufficient to counteract persistent damage, ultimately leading to ulcer formation (17). Persistent hyperglycemia also promotes local tissue hypoxia through microvascular dysfunction, which in turn exacerbates oxidative stress and activates inflammatory signaling pathways. The resulting oxidative damage further compromises endothelial integrity and disrupts the balance between vasodilatory and vasoconstrictive mediators, creating a self-perpetuating cycle of microcirculatory impairment (18). In the present study, patients with DFU exhibited elevated CRP levels and reduced albumin levels, suggesting that inflammatory activation and nutritional depletion may jointly contribute to disease progression in a hypoxic and oxidative microenvironment.

Among the three inflammatory biomarkers evaluated, CAR demonstrated the most stable association. One major advantage of CAR lies in its simultaneous integration of inflammatory status (CRP) and nutritional reserve (albumin). CRP, as a classical acute-phase reactant, has been widely used to reflect systemic inflammatory burden (19). In contrast, albumin not only reflects nutritional status but also possesses antioxidant and multiple biological functions. Reduced albumin levels may indicate inadequate nutritional reserve and are associated with impaired antioxidant defense capacity and heightened inflammatory consumption (20). Therefore, CAR may more comprehensively reflect dysregulation of the “inflammation–nutrition axis,” thereby improving risk discrimination for DFU.

Previous studies in other disease settings, particularly gastrointestinal malignancies, have suggested that composite inflammation–nutrition biomarkers incorporating CRP and albumin, such as the CALLY index, may outperform single biomarkers in reflecting inflammatory burden and nutritional status. This trend is generally consistent with the findings of the present study, although further validation across different disease populations remains necessary (21). In contrast, although NLR and SII or platelet-to-lymphocyte ratio (PLR) frequently demonstrate statistical significance in univariate analyses, their stability in multivariate models appears relatively limited. Previous studies have reported that NLR is associated with diabetic nephropathy, retinopathy, and peripheral neuropathy, whereas SII and PLR are associated with nephropathy and retinopathy but not with peripheral neuropathy, suggesting that these inflammatory markers may differ in their predictive profiles across diabetic complications ^(^22). In population-based settings, both NLR and SII have demonstrated prognostic value for cardiovascular disease and mortality among individuals with diabetes, with NLR generally showing more consistent predictive performance than SII (23). Nevertheless, although these markers are significantly associated with adverse outcomes, their overall discriminatory ability remains modest, indicating that inflammatory biomarkers alone may be insufficient to provide reliable clinical discrimination across heterogeneous diabetic populations (24).

This may be attributable to substantial variability in cutoff values among studies, lack of standardized thresholds, and susceptibility to influence from various physiological and pathological conditions (25)^.^ Although SII incorporates platelet parameters and may therefore improve sensitivity to inflammatory responses, its components primarily reflect acute immune-inflammatory activity and do not directly capture nutritional reserve (26). The inconsistency between univariate significance and limited multivariate stability may reflect the susceptibility of NLR and SII to acute physiological fluctuations—including concurrent infections, medication effects, and stress responses—which compromise their reliability as stable risk indicators across diverse clinical settings.

The present study further demonstrated that the final integrated model achieved an AUC of 0.84 in internal validation and significantly outperformed the other models. In independent external validation, the AUC was 0.61. The differences in DFU incidence between the two centers (16.7% vs. 34.0%) and variations in baseline patient characteristics may partially explain the decline in AUC observed during external validation. In addition, a prespecified sensitivity analysis excluding patients with moderate-to-severe DFU infection substantially attenuated the CAR association in both cohorts, indicating that CAR's performance is largely driven by acute infection-related inflammation rather than reflecting a stable predisposition to DFU. Nevertheless, DCA results consistently demonstrated favorable net clinical benefit across a broad threshold range, further supporting the potential clinical utility of Model 4 for risk stratification in hospital settings.

This study also has important clinical implications. CAR is derived from routine laboratory parameters and is inexpensive, convenient, and readily accessible in both inpatient and primary healthcare settings. The integrated risk model combining CAR with clinical variables may facilitate hospital-based cross-sectional risk stratification of hospitalized patients with T2DM, thereby guiding timely diagnostic evaluation and comprehensive risk assessment.

Several limitations should also be acknowledged. First, as a retrospective cross-sectional study, inherent selection bias and information bias could not be completely avoided. Moreover, the cross-sectional design precludes causal inference; elevated inflammatory markers could result from the ulcer itself rather than precede its development (reverse causation). Although statistical adjustment was performed, residual confounding may still have affected model performance. We therefore emphasize the need for randomized controlled trials or prospective cohort studies to establish clearer causal relationships. Second, the external validation cohort was derived from hospitals within the same geographical region, and multicenter validation across different regions and healthcare levels was lacking, which may limit the broader generalizability of the model. Third, only single-time-point laboratory measurements obtained at admission were included, and dynamic longitudinal changes in inflammatory and nutritional biomarkers were not evaluated. Therefore, the temporal evolution of the inflammation–nutrition status during disease progression could not be assessed. Future studies with repeated measurements at multiple time points are strongly encouraged to capture dynamic biomarker trajectories. Fourth, the observed CAR-DFU association was substantially attenuated after excluding patients with moderate-to-severe infection, indicating that infection-related inflammation is a major confounder; this limits the model's applicability to non-infected populations or community screening. Fifth, several clinically relevant variables, including diabetic nephropathy stage, specific categories and dosages of glucose- and lipid-lowering medications, lifestyle factors, and foot care behaviors, were not included. Notable cross-cohort differences in the direction of certain variables (e.g., smoking history, lymphocyte count) also suggest residual confounding that warrants further investigation. These factors have been recognized as important determinants of DFU risk and may contain additional prognostic information. We call for prospective collection of these variables in future research to further refine the model. Finally, neuropathy and PVD—although strongly associated with DFU—were excluded from the final model due to quasi-complete separation, meaning their predictive contribution could not be independently estimated in this cohort.

Overall, CAR is identified as independently associated with prevalent DFU in patients with T2DM, but its association is largely driven by acute infection-related inflammation. The integrated model combining CAR with clinical variables demonstrates good discrimination in internal validation and modest performance in independent validation after excluding neuropathy and PVD. These findings further emphasize the important contribution of inflammation and nutritional status to DFU pathophysiology and provide supportive evidence for cross-sectional risk stratification in hospitalized patients, although prospective studies in non-infected populations are needed before broader implementation.

## Conclusion

In this multicenter retrospective cross-sectional study, CAR was independently associated with prevalent DFU in hospitalized patients with T2DM, an association largely driven by acute infection-related inflammation. The integrated risk model combining CAR with clinical variables showed good internal discrimination and modest external performance, with miscalibration and threshold heterogeneity between cohorts indicating the need for local recalibration before clinical use. These findings support CAR's potential utility for cross-sectional risk stratification in hospital settings, but its role in prospective cohort studies in community-based populations in non-infected populations requires further investigation.

## Data Availability

The raw data supporting the conclusions of this article will be made available by the authors, without undue reservation.
