# Supplementary material for: Comparative screening performance and multicenter validation of novel inflammatory biomarkers (NLR, SII, and CAR) for risk stratification of diabetic foot ulcer in patients with type 2 diabetes mellitus
Source: Front Med (Lausanne). 2026 Sep 8;13:1890603. doi: 10.3389/fmed.2026.1890603 (PMC13593486; doi:10.3389/fmed.2026.1890603)
Supplement: Supplementary file 1 [file Table1.docx]

**Supplementary Figure S1.** Restricted cubic spline (RCS) analysis of the association between CAR and DFU in the Dujiangyan cohort.

The solid line represents the odds ratio, and the shaded area represents the 95% confidence interval. The histogram at the bottom shows the distribution of CAR values. The relationship between CAR and the log-odds of DFU was approximately linear (*P* for non-linearity > 0.05), supporting the linear specification of CAR in the multivariable logistic regression models.

**Supplementary Figure S2.** LASSO regression for variable selection in the Dujiangyan cohort.

(a) LASSO coefficient paths for the candidate variables. Each colored line represents the trajectory of a coefficient as the penalty parameter λ increases. The vertical dashed lines indicate λ.min (minimum cross-validation error) and λ.1se (one standard error from the minimum). (b) 10-fold cross-validation binomial deviance curves. The optimal λ (λ.1se) selected the most parsimonious model with robust performance.

**Supplementary Table S1.** Sensitivity analysis: Impact of excluding moderate-to-severe DFU infection on the association between CAR and DFU

| **Cohort** | **Analysis** | **CAR OR (95% CI)** | ***P*-value** | **AUC (95% CI)** | **Model** |
| --- | --- | --- | --- | --- | --- |
| **Dujiangyan (Training, n=962)** | Main analysis (all patients) | 1.331 (1.197–1.489) | <0.001 | 0.838 (0.803–0.872) | Model 4a (7 variables) |
|  | Sensitivity analysis (excluding moderate-to-severe infection) | 1.162 (0.962–1.402) | 0.12 | 0.803 (0.761–0.842) | Model 4a (7 variables) |
| **Fuling (External validation, n=600)** | Main analysis (all patients) | 1.139 (1.010–1.286) | 0.031 | 0.611 (0.563–0.660) | Model 4b (6 variables) |
|  | Sensitivity analysis (excluding moderate-to-severe infection) | 0.984 (0.798–1.214) | 0.84 | — | Model 4b (6 variables) |

Abbreviations: CAR, C-reactive protein-to-albumin ratio; OR, odds ratio; CI, confidence interval; AUC, area under the curve; DFU, diabetic foot ulcer. Note: ORs for CAR are per 0.1-unit increase. Model 4a included CAR + sex + age + creatinine + cholesterol + lymphocyte count + smoking history; Model 4b included CAR + sex + age + creatinine + lymphocyte count + smoking history (cholesterol unavailable in Fuling cohort). The sensitivity analysis excluded patients with moderate or severe infection from both cohorts, and multivariate logistic regression was repeated with the same covariate adjustments. —, not estimated due to insufficient sample size after exclusion.

**Supplementary Table S2.** Calibration metrics and Brier scores in the training and validation cohorts

| **Model** | **Dujiangyan (Training, n=962)** | | | | **Fuling (Validation, n=600)** | | | |
| --- | --- | --- | --- | --- | --- | --- | --- | --- |
|  | **Calibration-in-the-large** | **Calibration slope** | **Hosmer-Lemeshow (χ², *P*)** | **Brier** | **Calibration-in-the-large** | **Calibration slope** | **Hosmer-Lemeshow (χ², *P*)** | **Brier** |
| Model 1 | 0 | 1 | 5.1 (0.747) | — | 0.137 | 0.551 | 102.5 (<0.001) | — |
| Model 2 | 0 | 1 | 4.9 (0.773) | — | 0.179 | 0.595 | 93.2 (<0.001) | — |
| Model 3 | 0 | 1 | 3.4 (0.909) | — | −0.141 | 0.362 | 124.8 (<0.001) | — |
| Model 4 | −0.000 | 1 | 3.9 (0.866) | 0.0979 | −0.684 | −0.028 | 533.9 (<0.001) | 0.2127 |

Abbreviations: Brier, Brier score (lower indicates better calibration accuracy). Note: Calibration slope ideal = 1; calibration-in-the-large ideal = 0. Hosmer-Lemeshow test *P* > 0.05 indicates no significant lack of calibration. Brier scores for Models 1–3 in external validation are not presented separately; Model 4 values are derived from incremental analysis (see Table 4). Model 4 corresponds to Model 4a in Dujiangyan and Model 4b in Fuling. The marked external miscalibration (slope = −0.028, *P* < 0.001) reflects substantial case-mix and prevalence differences between the two cohorts.

**Supplementary Table S3.** Variable distribution and scaling rationale

| **Variable** | **Mean** | **SD** | **Median** | **P25** | **P90** | **Unit used** | **Rationale** |
| --- | --- | --- | --- | --- | --- | --- | --- |
| CAR | 0.698 | 1.368 | 0.25 | 0.23 | 1.399 | per 0.1 | IQR 0.23–0.31; 1-unit = 4× median |
| NLR | 6.272 | 8.154 | 3.58 | 2.38 | 12.463 | per 1 | SD=8.2; 1-unit clinically interpretable |
| SII | 1120.689 | 1756.817 | 583.615 | 371.897 | 2378.961 | per 100 | Median 584; 1-unit too small |
| Age | 63.641 | 14.326 | 65 | 54 | 81 | per 1 yr | Standard, clinical convention |
| Creatinine | 84.866 | 73.507 | 67.515 | 55.8 | 126.415 | per 10 | Median 67.5; 1-unit change negligible |
| Cholesterol | 4.447 | 1.349 | 4.33 | 3.57 | 5.999 | per 1 | Mean 4.45, SD 1.35; 1-unit reasonable |
| Lymphocyte count | 1.306 | 0.664 | 1.21 | 0.87 | 2.04 | per 1 | Mean 1.31, SD 0.66; 1-unit acceptable |

Abbreviations: SD, standard deviation; IQR, interquartile range; CAR, C-reactive protein-to-albumin ratio; NLR, neutrophil-to-lymphocyte ratio; SII, systemic immune-inflammation index.
